# Supplementary material for: Digital cognitive behavioural therapy for cardiac anxiety following acute coronary syndrome: protocol for a randomised controlled trial comparing CBT to a digital lifestyle intervention
Source: BMJ Open. 2025 Oct 28;15(10):e106473. doi: 10.1136/bmjopen-2025-106473 (PMC12570923; doi:10.1136/bmjopen-2025-106473)
Supplement: online supplemental file 1 [file bmjopen-15-10-s001.docx]

**Supplementary Material – Intervention Content**

**Supplementary Textbox. 1. Contents of CA-CBT**

| Module 1 - Introduction and psychoeducation | - Common emotional responses following ACS - The relationship between cardiac anxiety and avoidance behavior - Identifying avoidance and control behaviours - Goal-setting - Labeling Education on ACS, risk factors, its treatments and medication |
| --- | --- |
| Module 2 - Interoceptive exposure | - Exposure to physical sensations to reduce the associated fear - General lifestyle advice on e.g. physical activity |
| Module 3 - Introduction to exposure in-vivo | - Gradual exposure to avoided situations, places, and activities - Reduction of avoidance and control behaviours |
| Module 4 – Continuing exposure in more areas | - Rationale for exposure strategies to manage , e.g. worry, depression, fatigue, stress, and pain |
| Modules 5, 6, 7 - Continuing exposure and reclaiming activities | - Continuous work with gradual interoceptive and in-vivo exposure - Combining the treatment strategies |
| Module 8 - Summary and relapse prevention | - Summary of treatment - Identifying risk situations - Plan for future work towards goals |

| **Supplementary Textbox 2. Contents of CLI** | |
| --- | --- |
| Module 1 – About ACS, causes and treatment | - Education on ACS, risk factors, its treatments and medication - Rationale on how support in implementing lifestyle changes after ACS can reduce health-related worry and promote a sense of control. - Behavioural change strategies targeting health behaviours, such as goal setting, rewards, identifying barriers, stepwise progression, identifying short and long-term consequenses |
| Module 2 - Dietary habits, alcohol and tobacco | - Education and advice promoting healthy habits regarding diet, alcohol and tobacco - Behavioural strategies related to the areas |
| Module 3 - Physical activity | - Education regarding physical activity and the beneficial effects on health - Behavioural strategies related to physical activity |
| Module 4 - Common emotional reactions | - Education regarding common emotional reactions following ACS - Tools related to emotional reactions such as…. |
| Module 5, 6, 7 – Continued work with relevant areas | - Repetition of the relevant information and behavioural strategies from previous modules |
| Module 8 - Maintain a healthy lifestyle | - Prevention of relapse and plan forward to maintain a healthy lifestyle |
