# Supplementary material for: Digital cognitive behavioural therapy for cardiac anxiety following acute coronary syndrome: protocol for a randomised controlled trial comparing CBT to a digital lifestyle intervention
Source: BMJ Open. 2025 Oct 28;15(10):e106473. doi: 10.1136/bmjopen-2025-106473 (PMC12570923; doi:10.1136/bmjopen-2025-106473)
Supplement: Supplementary file 4 [file bmjopen-2025-106473.reviewer_comments.pdf]

PEER REVIEW HISTORY

BMJ Open publishes all reviews undertaken for accepted manuscripts. Reviewers are asked to complete a checklist review form and are provided with free text boxes to elaborate on their assessment. These free text comments are reproduced below.

ARTICLE DETAILS

Title (Provisional)

Digital Cognitive Behavioral Therapy for Cardiac Anxiety Following Acute Coronary Syndrome: Protocol for a Randomised Controlled Trial Comparing CBT to a Digital Lifestyle Intervention

Authors

Johnsson, Amanda; Ljótsson, Brjánn; Braunschweig, Frieder; Mellbin, Linda G; Särnholm, Josefin

---

VERSION 1 - REVIEW

**Reviewer**	**1**
**Name**	**Deter, Hans-Christian**
**Affiliation**	**Psychosomatics, Charité CBF, Medical Clinic, Division of Psychosomatic Medicine**
**Date**	**21-Aug-2025**
**COI**	**None**

Digital cognitive behavioral therapy for cardiac anxiety (CA-CBT) after acute coronary syndrome: Protocol for a randomized controlled trial comparing CBT with a digital lifestyle intervention (CLI) by A. Johnsson et al. is a clinically important paper that outlines a study protocol for an RCT comparing digital CA-CBT with digital CLI-CBT. This ongoing study will be completed next year, and the study concept is now to be published in BMJ Open. This study was supported by the Swedish Heart and Lung Foundation and the Swedish Research Council.

A. The background and concept of the study are presented clearly and plausibly and are supported by actual references. Specific cardiac anxiety symptoms in patients with a history of myocardial infarction (MI) or unstable angina pectoris (UA) should be treated with a specific treatment method that focuses on exposure (CA-CBT) and is compared with a control group that receives digital education and lifestyle changes and provides psychological support.

It is not clear to me what the reasons and causes of anxiety are in the different individuals—only 30% scored high on the Cardiac Anxiety Questionnaire (CAQ≥18) and in clinical

interviews. What is the cause of anxiety in the other individuals with CAD, as measured, for example, by the DSM expert assessment or other anxiety measures (HADS)?

B. I have several concerns about the methods used:

1. Can the authors control the selection of digital invitations in Sweden? What kind of patients would be interested in this extensive and time-consuming study without adequate compensation?

2. I found the topic of sex and gender to be lacking in the study. There is a higher correlation between anxiety and women compared to men. In addition, there are different responses between men and women in relation to psychosocial interventions. Why was this topic neglected?

3. The main hypothesis of the study was a clear advantage for CA-CBT, measured by a greater difference between the groups before and after therapy (which is very short), but not before and after the 6-month follow-up as in the secondary analyses. In my opinion, a before-and-after comparison is not acceptable for a study statement.

4. The study sample consisted of CAD patients 6 months after MI or UA. Did the authors verify this information through the Swedish health information system, and how long was the period in the patients' medical history? Did the event occur 6 months ago or 10 years ago? This makes a difference for cardiac anxiety.

Heart failure is important for the prognosis of CAD patients. Anxiety, coping strategies, and denial depend on the chance of a longer life. An EF threshold of <30% is very low and can also influence anxiety levels between 30% and 50% via B-type natriuretic peptide (Fangauf 2019).

However, patients with severe mental illness were excluded, but not all anxiety diagnoses were included in this chapter, so patients were included in the sample. If there is an individual reason for the development of anxiety, to what extent can the treatment method used here be tailored to it? Many cardiovascular events among study participants? High dropout rate – more than 26 patients?

5. The study is not subject to formal review. Are there criteria for discontinuing the study? How many cardiovascular events among study participants? High discontinuation rate – more than 26 patients?

6. Is the study activity implemented in the Swedish digital health system, and do general practitioners or cardiologists know about the activities in the study, especially in an emergency?

7. The study staff is not blinded. During treatment, there are several interactions with patients, psychologists, and the study nurse if the patient tends to drop out of the study in order to keep them in the study. This may influence the study

Discussion

The denial of anxiety is sometimes an important aspect in clinical practice. Perhaps the authors could include this in the discussion.

The possible limitations of the study results should be presented more clearly at the end of the discussion.

VERSION 1 - AUTHOR RESPONSE

Reviewer: 1
Dr. Hans-Christian Deter, Psychosomatics, Charité CBF
Comments to the Author:
Digital cognitive behavioral therapy for cardiac anxiety (CA-CBT) after acute coronary syndrome: Protocol for a randomized controlled trial comparing CBT with a digital lifestyle intervention (CLI) by A. Johnsson et al. is a clinically important paper that outlines a study protocol for an RCT comparing digital CA-CBT with digital CLI-CBT. This ongoing study will be completed next year, and the study concept is now to be published in BMJ Open. This study was supported by the Swedish Heart and Lung Foundation and the Swedish Research Council.

Response:
Thank you for summarizing the study and acknowledging its clinical importance. We appreciate your recognition of the study's relevance and the support from the Swedish Heart and Lung Foundation and the Swedish Research Council.

Reviewer comment:
A. The background and concept of the study are presented clearly and plausibly and are supported by actual references. Specific cardiac anxiety symptoms in patients with a history of myocardial infarction (MI) or unstable angina pectoris (UA) should be treated with a specific treatment method that focuses on exposure (CA-CBT) and is compared with a control group that receives digital education and lifestyle changes and provides psychological support.
It is not clear to me what the reasons and causes of anxiety are in the different individuals—only 30% scored high on the Cardiac Anxiety Questionnaire (CAQ≥18) and in clinical interviews. What is the cause of anxiety in the other individuals with CAD, as measured, for example, by the DSM expert assessment or other anxiety measures (HADS)?

Response:

Thank you for your comment and for highlighting the importance of clarifying the study population. The prevalence figures in the introduction refer to the general post-ACS

population and not to the study sample. As described in the Methods section, this trial exclusively includes patients with clinically significant cardiac anxiety (CAQ ≥18). The study is ongoing, and no data on individual causes of anxiety are available at this stage. We have revised the sentence in the introduction to avoid misunderstanding. The revised sections are marked in red

P 5 Revised sentence:

Previous studies indicate that approximately one third of patients develop clinically significant cardiac anxiety following ACS (1).

Reviewer comment:
B. I have several concerns about the methods used:
1. Can the authors control the selection of digital invitations in Sweden? What kind of patients would be interested in this extensive and time-consuming study without adequate compensation?

Response:

Thank you for raising this important point. Recruitment is conducted nationwide via multiple channels: (i) broad public advertisements (social media, daily press) and (ii) postal invitations based on medical records from a tertiary cardiac center. While we cannot control the internal delivery algorithms of social media platforms, we mitigate potential selection bias by using standardized invitations, disseminating them across several channels, and supplementing digital outreach with record-based postal invitations. Eligibility is then determined through a structured, multi-step screening and clinical assessment process, as detailed in the Methods, and the CONSORT flow (Figure 1) will document the number screened, excluded, and randomized.

Regarding motivation to participate without monetary compensation, all participants experience clinically significant cardiac anxiety and currently lack access to structured psychological treatment for this condition in routine care. Thus, participation offers an opportunity to receive a disease-specific therapist-supported digital intervention, which many patients perceive as valuable. The interventions are delivered remotely, reducing practical burden, and participants may withdraw at any time. Our previous studies on CA-CBT have shown high adherence with over 80% of the participants completing the treatment (2).

Additionally, our institutional policy is to reimburse study participants for out-of-pocket expenses such as travel and parking; however, this is not applicable in the present on-line study. While financial compensation for time and effort can be considered reasonable, it also carries the risk of motivating participation primarily for economic reasons, which we wished to avoid. Importantly, as previously mentioned, our previous

studies on CA-CBT have demonstrated high adherence, with more than 80% of participants completing the treatment, suggesting that additional monetary incentives are not necessary to ensure retention.

We have clarified these aspects in the manuscript and added a section in the discussion (see suggested edits below).

P 11 Added sentence:

All study activities are conducted remotely to reduce participant burden (no on-site visits), and automated reminders plus follow-up contacts support timely completion of assessments.

P23 Added sentence:

No monetary compensation is provided; participation involves access to an eight-week, therapist-supported digital intervention in either study arm for individuals experiencing clinically significant cardiac anxiety following ACS.

Reviewer comment:
2. I found the topic of sex and gender to be lacking in the study. There is a higher correlation between anxiety and women compared to men. In addition, there are different responses between men and women in relation to psychosocial interventions. Why was this topic neglected?
Response:

Thank you for this important comment. We agree that sex and gender differences are highly relevant in the context of cardiac anxiety and psychosocial interventions. In the current trial, we did not include sex or gender as stratification factors in the randomisation process, nor was the study powered to detect interaction effects by sex. However, we will report the sex distribution and describe the characteristics of cardiac anxiety and QoL in men and women at baseline as well as keeping sex and gender considerations in mind when interpreting the results.

Reviewer comment:
3. The main hypothesis of the study was a clear advantage for CA-CBT, measured by a greater difference between the groups before and after therapy (which is very short), but not before and after the 6-month follow-up as in the secondary analyses. In my opinion, a before-and-after comparison is not acceptable for a study statement.
Response:

Thank you for this valuable comment and the opportunity to clarify. The primary analysis is not based on a simple before-and-after comparison. Instead, we will use a linear mixed-effects model including all repeated measures during the treatment period (baseline, eight weekly assessments, and post-intervention). This approach accounts for the entire trajectory of change and is more robust than a single pre-post comparison.

The primary endpoint was chosen as post-intervention to capture the immediate treatment effect, which is the main objective of the trial. Longer-term maintenance of effects will be examined in secondary analyses at 6 and 12 months. This approach is consistent with common practice in psychological intervention trials and aligns with CONSORT recommendations, where the primary endpoint typically reflects the immediate treatment effect.

We agree that in general, the main outcomes of interests in a behavioral intervention are the long-term effects. However, the primary purpose of the present study is to control for non-specific effects of a behavioral treatment intervention. We expect substantial improvements in the active control group and have therefore selected an analytic method that maximizes statistical power. After the acute treatment phase has ended, other factors will probably affect the development on the main outcomes and dilute potential differences between CA-CBT and CLI. In a scenario where we observe a statistically significant treatment effect of CA-CBT in the main analysis but that this difference diminishes at 6MFU or 12MFU, this could be due to ”sleeper effects” in the CLI arm, i.e., reflecting that CLI patients catch up over a longer time. But we have only powered the study to detect differences in acute true treatment effects, and would need a considerably larger sample if we wanted to compare single-point assessments at 6MFU or 12MFU between the groups. In essence, we agree that we can only determine whether the effects of CA-CBT are due to non-specific factors in the acute treatment phase. This limitation will be addressed when reporting the trial results.

To avoid misunderstanding, we have slightly revised the text in the Objectives section to clarify this point. The revised sections are marked in red.

P 7 rephrased section:

We hypothesise that exposure-based digital CBT targeting cardiac anxiety following ACS is effective in reducing cardiac anxiety and depressive symptoms and improving disease-specific QoL and physical activity over the course of the treatment period (i.e. baseline, weekly measures over eight weeks, post-intervention). We further anticipate that these improvements will be maintained at six- and twelve-month follow-up, which will be examined as secondary outcomes.

Reviewer comment:

4. The study sample consisted of CAD patients 6 months after MI or UA. Did the authors verify this information through the Swedish health information system, and how long was the period in the patients' medical history? Did the event occur 6 months ago or 10 years ago? This makes a difference for cardiac anxiety.

Response:

Thank you for this important question. All participants' ACS events were verified through their medical records before inclusion. In previous studies, we have observed that the time since the ACS event varies considerably among patients with elevated cardiac anxiety. However, research and clinical experience indicate that cardiac anxiety can persist as a stable and clinically significant condition over time, continuing to affect daily life long after the initial event.

To ensure clinical relevance, all participants undergo a structured clinical assessment prior to inclusion to confirm that they meet criteria for cardiac anxiety, rather than a more general anxiety disorder. We will report the time since the index ACS event descriptively in the baseline characteristics table.

To address your question, we have clarified the procedures regarding this and revised the section. The revised text is marked in red.

P 9-10 revised section:

Following the screening, a chart review is conducted by the cardiac study nurse to verify the ACS diagnosis and classification (MI or unstable angina), confirm the date of the index event, and review comorbid conditions, medical history and key cardiac parameters, including recent blood pressure, lipid status, ECG, echocardiogram results (if available), and current medications. This verification is based on the patient's medical records. Thereafter, the nurse proceeds with a telephone assessment to confirm medical history and collect additional cardiac parameters. Eligible applicants then undergo a telephone-based psychological assessment by a study psychologist, to confirm clinically significant cardiac anxiety and to exclude severe psychiatric disorders, including severe depression, risk of suicide and/or alcohol dependency. This assessment ensures that cardiac anxiety is the primary clinical concern rather than other psychological distress. The medical assessments and the cardiac parameters from the medical records for eligible applicants are then reviewed by the study cardiologist to determine whether the participant meets the eligibility criteria.

Reviewer comment:

Heart failure is important for the prognosis of CAD patients. Anxiety, coping strategies,

and denial depend on the chance of a longer life. An EF threshold of <30% is very low and can also influence anxiety levels between 30% and 50% via B-type natriuretic peptide (Fangauf 2019)
However, patients with severe mental illness were excluded, but not all anxiety diagnoses were included in this chapter, so patients were included in the sample. If there is an individual reason for the development of anxiety, to what extent can the treatment method used here be tailored to it? Many cardiovascular events among study participants? High dropout rate – more than 26 patients?

Response:

Thank you for these important points. We agree that cardiac function and heart failure status are clinically relevant factors that may influence prognosis and psychological responses. In our study, EF and other cardiac parameters are verified through medical records during the eligibility assessment. Patients with EF <30% or NYHA class IV are excluded to ensure clinical safety and feasibility of participation. EF will be reported descriptively in the baseline characteristics table.

Regarding mental health, patients with severe mental illness are excluded, which can include severe anxiety disorders if the psychiatric burden is judged to require treatment prior to addressing cardiac anxiety. Psychological distress often involves overlapping symptoms, and some participants may present with other anxiety features. However, all participants undergo a structured psychological assessment to confirm that cardiac anxiety is clinically significant and to assess weather concurrent mental health conditions that would either hinder participation or require other treatment intervention or clinical attention are identified. Of note, presence of an anxiety disorder (for example panic disorder or social anxiety disorder) will typically not lead to exclusion, because in most cases these diagnoses are not considered to require immediate treatment or to be obstacles to completing CA-CBT. However, presence of eating disorders or untreated bipolar disorder are more likely to lead to exclusion.

The CA-CBT protocol is manual-based but allows for individual tailoring within the framework of the treatment. Therapists adapt examples and exercises to the participant's specific fears and life context while maintaining fidelity to the treatment model.

Cardiovascular events are monitored throughout the intervention period, and to date, no events have been reported. This is consistent with our previous pilot studies and RCT, where no adverse cardiac events occurred during treatment. As the trial is ongoing, dropout rates are not yet available, but these will be transparently reported in the final publication, including reasons for discontinuation where possible.

Regarding drop-out, we assume the reviewer is referring to the power calculation. We based our sample size calculation on an expected attrition rate of 15%, which is conservative in the context of internet-delivered CBT. Meta-analyses consistently report dropout rates of 20–30% across disorders. Our intervention includes therapist guidance, structured reminders, and early engagement strategies, all of which have been shown to improve adherence. Therefore, we anticipate a lower attrition rate than typically observed in the literature and consider 15% a reasonable and conservative assumption for powering this trial.

Reviewer comment:
5. The study is not subject to formal review. Are there criteria for discontinuing the study? How many cardiovascular events among study participants? High discontinuation rate – more than 26 patients?

Response:

Thank you for this comment and the opportunity to clarify. The study protocol was reviewed and approved by the Swedish Ethical Review Authority (Dnr 2023-07605-01) and registered at ClinicalTrials.gov (NCT06298864). While there is no independent data monitoring committee or predefined stopping rules for the entire study, this is due to the low-risk nature of the intervention and the extensive safety measures in place. The intervention content has been reviewed by cardiologists, and previous trials using the same approach have reported no serious adverse events.

As described in the Safety section, participants undergo thorough pre-inclusion assessments by both a cardiac nurse and a cardiologist to ensure that participation poses no medical risk. Safety is continuously monitored during the intervention, and any participant with symptoms requiring medical attention is referred to standard care. To date, no cardiovascular events have occurred among participants, which is consistent with our previous studies (2, 3).

Regarding discontinuation rates, the sample size calculation accounted for expected attrition to maintain adequate power. As the trial is ongoing, final dropout numbers are not yet available, but these will be transparently reported in the results, including reasons for discontinuation where possible.

Reviewer comment:
6. Is the study activity implemented in the Swedish digital health system, and do general practitioners or cardiologists know about the activities in the study, especially in an emergency?

Response:

Thank you for this important question. The study is not implemented as part of any national Swedish digital health platform, but participation is documented in the patient's electronic medical record within the healthcare system. This ensures that treating physicians, including general practitioners and cardiologists, can access this information if needed, for example in an emergency situation. Participants are informed that their regular physician remains responsible for their medical care and that any questions or concerns regarding medication or cardiac symptoms should be directed to their healthcare provider. The intervention consists of psychological strategies and behavioural exercises aligned with standard cardiac rehabilitation recommendations and does not introduce additional medical risk. To clarify these procedures, we have revised and added a description in the Methods section under "Safety and Risk Management." The revised sections are marked in red

P 15-16 Revised section:

No additional medical risks are anticipated from participation in the trial, as the intervention aligns with post-ACS recommendations. The content has been reviewed and approved by cardiologists, and previous studies on CA-CBT have reported no serious adverse events (23) (Johnsson et al., manuscript in preparation; ClinicalTrials: NCT05580718). Several essential safety measures are in place, including a pre-inclusion assessment by a cardiac nurse and cardiologist to ensure that participation poses no risk to the participants' safety, and that the applicant has no contraindication to be physically active. In addition to these medical safety procedures, psychological safety is also addressed: Structured psychological assessments are conducted before inclusion, and applicants with high-risk suicidal ideation or other severe conditions are referred to standard care.

During the interventions, psychologists have on-demand access to supervision from a cardiac study nurse and cardiologist through the BASS platform during the interventions, particularly for issues related to reported symptoms or the adaptation of exercises to the participant's health status. Study participation is documented in the electronic medical record, ensuring accessibility for treating physicians. Participants are instructed to contact their regular healthcare provider for any medical concerns. Both intervention groups receive guidance on when to seek medical care and how to distinguish benign from acute cardiac symptoms (e.g., palpitations during exercise vs. persistent chest pain). Participants also self-report unwanted effects (28) weekly during the study, and at follow-up, to monitor adverse events.

Reviewer comment:
7. The study staff is not blinded. During treatment, there are several interactions with patients, psychologists, and the study nurse if the patient tends to drop out of the study in order to keep them in the study. This may influence the study

Response:

Thank you for this important observation. We acknowledge that blinding of study staff is not feasible in psychological intervention trials, as therapists must deliver the treatment and interact with participants. To minimize potential bias, outcome assessments are based entirely on self-reported questionnaires completed by participants online, without study personell involvement in data collection.

Regarding retention, participants receive standard reminders to complete modules and assessments, which is common practice in digital interventions to reduce attrition. These reminders are automated or follow a structured protocol and do not involve therapeutic content beyond what is specified in the intervention manuals. Importantly, these procedures are applied equally across both study arms to maintain consistency.

Reviewer comment:
Discussion
The denial of anxiety is sometimes an important aspect in clinical practice. Perhaps the authors could include this in the discussion.
Response:

Thank you for this suggestion. BMJ Open protocol articles do not include a traditional discussion section, and we therefore limit the manuscript to methodological and procedural aspects. For this reason, we have not added a discussion of clinical phenomena such as denial of anxiety. However, we acknowledge its relevance and will consider addressing this aspect when reporting the trial results.

That said, we fully acknowledge the clinical relevance of anxiety denial and agree that it may play an important role in how patients engage with psychological interventions. In our study, the psychological interview includes questions about behavioral changes following myocardial infarction, which may help identify avoidance patterns. We also use broad and inclusive terminology—such as “worry” rather than strictly “anxiety”—to support engagement and reduce the risk of alienating participants who may not identify with psychiatric labels.

Reviewer comment:
The possible limitations of the study results should be presented more clearly at the end

of the discussion.

Response:

Thank you for this comment. In accordance with BMJ Open protocol guidelines, we present study limitations in the Strengths and limitations of this study section rather than in a separate discussion. We have revised this section to make the limitations more explicit.

To address your comment, we clarified the limitations in the bullet points after the abstract, for example by highlighting potential issues related to generalisability, reliance on self-report measures, and the overlap of behavioural components between study arms.

P 4 revised bullet points:

Strengths and limitations

- An active control group is used to account for non-specific effects, although both interventions include behavioural components, which may reduce between-group contrast.
- Physical activity is measured objectively with accelerometry alongside validated self-report instruments; however, reliance on self-report for several secondary outcomes may still introduce reporting bias.
- Study procedures incorporate a multidisciplinary assessment and a collaborative approach to treatment delivery, and both interventions are delivered as manual-based treatments with therapist support to maintain protocol adherence while allowing individualisation.
- Inclusion criteria (ACS ≥ 6 months and clinically significant cardiac anxiety) ensure safety and target the intended population, but findings may not generalise to ACS patients without cardiac anxiety or those in the acute phase of recovery.
- Therapists deliver both interventions across study arms to minimise therapist-related effects, supported by training and supervision.

1. Hohls JK, Beer K, Arolt V, Haverkamp W, Kuhlmann SL, Martus P, et al. Association between heart-focused anxiety, depressive symptoms, health behaviors and healthcare utilization in patients with coronary heart disease. Journal of Psychosomatic Research. 2020;131:109958.
2. Särnholm J, Skúladóttir H, Rück C, Axelsson E, Bonnert M, Bragesjö M, et al. Cognitive Behavioral Therapy Improves Quality of Life in Patients With Symptomatic Paroxysmal Atrial Fibrillation. Journal of the American College of Cardiology. 2023;82(1):46–56.

3. Johnsson A, Ljótsson B, Liliequist BE, Skúladóttir H, Maurex L, Boberg I, et al. Cognitive Behavioural Therapy targeting Cardiac Anxiety Post-Myocardial Infarction: Results from Two Sequential Pilot Studies. European Heart Journal Open. 2025.

VERSION 2 - REVIEW

Reviewer 1

Name Deter, Hans-Christian

Affiliation Psychosomatics, Charité CBF, Medical Clinic, Division of Psychosomatic Medicine

Date 09-Oct-2025

COI

The article has been significantly improved and, in my opinion, is now ready for publication.
